# 

**Published:** 1880-06

**Authors:** 


### Table of Contents.

### ORIGINAL COMMUNICATIONS.

A Case of Stone in the Bladder. By A. B. Copeland, M D., Hamilton, Ga ..... 129

Report of a Case of Intussusception of the Bowel. By N. B. Kennedy, M D., Hillsboro, Texas ..... 131

The Objections to the Metric System By C. W. Erwin, Allendale, S C ..... 133

### REPORTS OF SOCIETIES.

Obstetric Section of New York Academy of Medicine ..... 135
New York Academy of Medicine ..... 140
New York Pathological Society ..... 144
Cincinnati Medical Society ..... 147
Academy of Medicine ..... 154
Medical Association of Delaware County, (Ohio) ..... 157

### SELECTIONS.

Sterility in Women ..... 159

### EDITORIAL.

Apology ..... 168
Editorial Correspondence ..... 168
Quarantine and National Board of Health ..... 172
American Medical Association—Proceedings of General Session ..... 176
Meteorological Report for May ..... 185

### BIBLIOGRAPHICAL

Homeopathy: What Is It? ..... 186
The Management of Children in Sickness and in Health ..... 187
Health and Healthy Homes ..... 187

### EXCERPTA.

Bromide of Ethyl ..... 188
Catarrh of the Bladder ..... 189
Spray in Antiseptic Surgery ..... 191
A New Method of Administering Kousso ..... 192

